# Substantial reprogramming of the *Eutrema salsugineum* (*Thellungiella salsuginea*) transcriptome in response to UV and silver nitrate challenge

**DOI:** 10.1186/s12870-015-0506-5

**Published:** 2015-06-12

**Authors:** Stefanie Mucha, Dirk Walther, Teresa M Müller, Dirk K Hincha, Erich Glawischnig

**Affiliations:** Lehrstuhl für Genetik, Technische Universität München, D-85354 Freising, Germany; Max-Planck-Institut für Molekulare Pflanzenphysiologie, 14476 Potsdam, Germany

**Keywords:** *Eutrema salsugineum*, *Thellungiella salsuginea*, Transcriptomics, Glucosinolate biosynthesis, Phytoalexin

## Abstract

**Background:**

Cruciferous plants synthesize a large variety of tryptophan-derived phytoalexins in response to pathogen infection, UV irradiation, or high dosages of heavy metals. The major phytoalexins of *Eutrema salsugineum* (*Thellungiella salsuginea*), which has recently been established as an extremophile model plant, are probably derivatives of indole glucosinolates, in contrast to Arabidopsis, which synthesizes characteristic camalexin from the glucosinolate precursor indole-3-acetaldoxime.

**Results:**

The transcriptional response of *E. salsugineum* to UV irradiation and AgNO_3_ was monitored by RNAseq and microarray analysis. Most transcripts (respectively 70% and 78%) were significantly differentially regulated and a large overlap between the two treatments was observed (54% of total). While core genes of the biosynthesis of aliphatic glucosinolates were repressed, tryptophan and indole glucosinolate biosynthetic genes, as well as defence-related *WRKY* transcription factors, were consistently upregulated. The putative *Eutrema WRKY33* ortholog was functionally tested and shown to complement camalexin deficiency in *Atwrky33* mutant.

**Conclusions:**

In *E. salsugineum*, UV irradiation or heavy metal application resulted in substantial transcriptional reprogramming. Consistently induced genes of indole glucosinolate biosynthesis and modification will serve as candidate genes for the biosynthesis of *Eutrema*-specific phytoalexins.

**Electronic supplementary material:**

The online version of this article (doi:10.1186/s12870-015-0506-5) contains supplementary material, which is available to authorized users.

## Background

The synthesis of bioactive compounds for adaptation to abiotic stress conditions and for defence against herbivores and pathogen infections is a fundamental survival strategy of plants. The biosynthesis of phytoalexins, which contain an indole moiety substituted with additional ring systems or side chains, often containing sulphur and nitrogen, is characteristic for cruciferous plants [1]. The individual structures are very diverse even among different *Brassica* cultivars. In *Arabidopsis thaliana,* a variety of compounds are synthesized from the intermediate indole-3-acetonitrile (IAN) in response to pathogen infection or heavy metal stress [2,3] with camalexin as the most prominent metabolite. The camalexin biosynthetic pathway from tryptophan and glutathione and its role in defence against a number of fungal pathogens has been investigated in detail [4]. Phytoalexin biosynthesis is induced upon pathogen infection, but also under harsh abiotic conditions, such as high dosages of heavy metal ions or UV light, which lead to the generation of reactive oxygen species and ultimately to programmed cell death. For studies on plant metabolism, abiotic stress treatments provide the advantage that no interference of pathogen metabolism, which is often strain specific [5], has to be taken into account.

*Eutrema salsugineum* has been established recently as an alternative model system for crucifers in addition to *Arabidopsis*, because of its high tolerance of various abiotic stresses [6]. The *E. salsugineum* genome sequence [7,8], as well as a reference transcriptome, [9] are available and additional transcriptomics data were published recently [8,10]. *E. salsugineum* is also referred to as *Thellungiella salsuginea*. The ecotype Shandong analysed in this study was initially assigned as *T. halophila* and this species name was used in a number of publications [11-13]. Consequently, gene and transcript sequences isolated from Shandong ecotype have been deposited under the species names *T. halophila*, *T. salsuginea* and *E. salsugineum*. According to work by Koch and German [14], the species name *T. salsuginea* is acceptable, but *E. salsugineum*, which we refer to in this manuscript, is preferred.

Within the Brassicaceae, *Eutrema* and *Arabidopsis* are rather distantly related and their last common ancestor is estimated to have lived 43 million years ago [8]. Still, large stretches of syntenic regions were identified in the genomes, allowing clear assignment of putative orthologs [7,8]. At the protein level, for the number of best hit pairs between *Eutrema* and *Arabidopsis* a peak at 85% amino acid sequence identity was determined [8].

*Eutrema* and *Arabidopsis* have developed a diversified spectrum of defence compounds, such as glucosinolates [11,15,16] and indolic phytoalexins. In *Arabidopsis*, these phytoalexins are predominantly synthesized from the intermediate indole-3-acetaldoxime [2,17], while the characteristic *Eutrema* phytoalexins are most likely derivatives of 1-methoxy-indole glucosinolate [18]. The identification of biosynthetic genes for presumably glucosinolate-derived (*Eutrema*) and glucosinolate-independent (*Arabidopsis*) phytoalexins will build the basis for metabolic engineering studies of indolic phytoalexins and for establishment of a model for phytoalexin evolution in the Brassicaceae.

In this work, we analysed the transcriptional reprogramming of *E. salsugineum* in response to abiotic stress conditions, which lead to the accumulation of phytoalexins. We show that genes of tryptophan and indole glucosinolate biosynthesis and modification are highly upregulated providing candidates for phytoalexin biosynthesis. Also the *Eutrema* ortholog of *WRKY33*, a key regulator of *Arabidopsis* phytoalexin induction, was highly upregulated, even though known WRKY33 target genes, such as *CYP71B15* [19] are apparently missing in *E. salsugineum.*

## Results and Discussion

### Induction of phytoalexin biosynthesis in response to UV light and silver nitrate spraying

The biosynthesis of phytoalexins by Brassicaceae species is induced by pathogen infection, but also specific abiotic stress treatments, such as high dosages of heavy metals and UV light. Applying abiotic stressors provides the advantage of a high degree of experimental reproducibility and excludes the modulation of plant defence reactions and metabolism by the pathogen. Induction of phytoalexin biosynthesis by the heavy metal salt CuCl and UV treatment was previously established by Pedras and coworkers [12,13]. Here, wasalexin induction was confirmed for 10-week old *E. salsugineum* (Shandong) leaves in response to UVC light, silver nitrate application, and *Botrytis cinerea* infection (Additional file 1: Figure S1).

**Figure 1:**
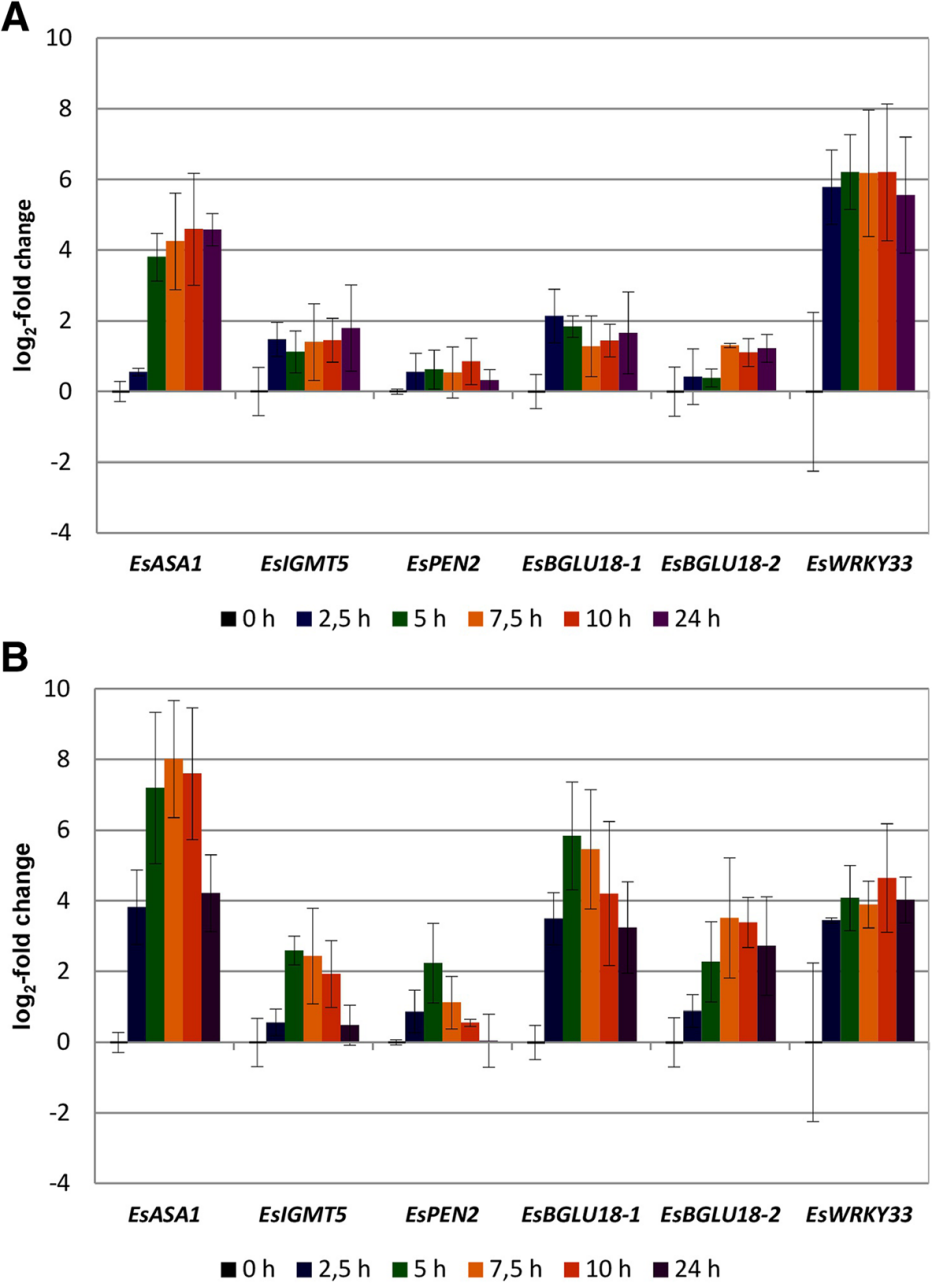
RT-qPCR analysis. Time course of expression after treatment with UV light **(A)** and AgNO_3_ **(B)**. *EsASA1 (Thhalv10013041m), EsIGMT5 (Thhalv10018739m), EsPEN2 (Thhalv10001354m), EsBGLU18-1 (Thhalv10011384m), EsBGLU18-2 (Thhalv10011385m), and EsWRKY33 (Thhalv10016542m)*, were analysed. The expression levels, relative to the mean for 0 h, were determined by RT-qPCR, normalized to the geometric mean of three reference genes (*EsActin1*, *EsYLS8* and *EsPP2AA2*). Values are means of three independent experiments ± SE.

In *Arabidopsis*, expression of camalexin biosynthetic genes is coregulated with expression of *ASA1*, encoding the committing enzyme of tryptophan biosynthesis. We therefore assumed that also in *E. salsugineum* tryptophan biosynthesis is upregulated under phytoalexin inducing conditions, which we later confirmed (see below). Quantitative RT-PCR was used to determine the induction kinetics of *EsASA1* (Figure 1). For both treatments, transcript levels were highly elevated 7.5 h and 10 h after the onset of induction. Therefore, for transcriptomics analysis 8 h induction was selected.

### The *Eutrema* transcriptome in response to UV light and heavy metal stress

RNA was isolated from non-treated leaves and from leaves treated with either AgNO_3_ or UV light. cDNA libraries were prepared and approximately 33 Mio to 45 Mio 50 bp reads per library were obtained by Illumina sequencing. Reads were mapped to the JGI genome [8]. For each cDNA library, approx. 75% of total transcript models were covered (Table 1) and a large overlap between treatments was observed (Additional file 2: Figure S2). Transcript models were analysed for read-counts in the different samples and annotated for best hit in the *Arabidopsis thaliana* genome (Additional file 3: Table S1).

**Table 1:** **RNAseq metrics and alignments**

		**n.i.**	**UV**	**AgNO**_**3**_	***B.c.***
reads	total fragments	33,445,682	45,326,703	33,278,110	35,924,995
	uncounted	8,100,893	22,525,573	7,470,863	12,091,407
	counted	25,344,789	22,801,130	25,807,247	23,833,588
	- uniquely	17,567,426	14,322,990	19,065,875	16,287,764
	- non-specific	7,777,363	8,478,140	6,741,372	7,545,824
transcripts	hit (reads > 0)	23,237	23,730	23,985	23,655
	uniquely hit	21,589	21,875	22,216	22,048
	(% of total)	(73,7%)	(74,7%)	(75,9%)	(75,3%)

Reads were mapped to the JGI genome (Yang et al., [8]), 29284 reference transcripts (2 mismatches allowed); uncounted/counted: number of unmapped/mapped reads; uniquely: number of uniquely mapped reads; non-specific: number of reads with multiple locations in the reference.

Similarly, we have analysed the transcriptome 48 h after infection of plants with *B. cinerea* (Additional file 3: Table S1). 3139 transcripts were identified as more than 2-fold upregulated with respect to untreated leaves. Of this set, 56% and 61% were also upregulated more than 2-fold after UV and AgNO_3_ treatment, respectively, indicating overlapping responses to the abiotic and biotic stressors. However, as transcriptional changes in response to UV light and AgNO_3_ were much more pronounced, we focussed on these treatments for further analysis.

Microarray analysis of four biological replicates was conducted with Agilent arrays based on the design by Lee et al. [9]. Statistically robust differential regulation was observed for the majority of transcripts (Additional file 4: Table S2). Of a total of 42562 oligonucleotide probes, signal intensities of 11930 (28%) and 15384 (36%) probes were significantly (t-test FDR corrected p < 0.01) elevated, while signal intensities of 11562 (27%) and 11879 (28%) probes were significantly reduced in response to UV light and AgNO_3_, respectively.

These array data were compared with the RNAseq data, which in addition provide information about absolute expression levels. A correlation analysis with the log_2_ fold-change values obtained by the two methods in response to UV and AgNO_3_ is shown in Additional file 5: Figure S3.

**Figure 2:**
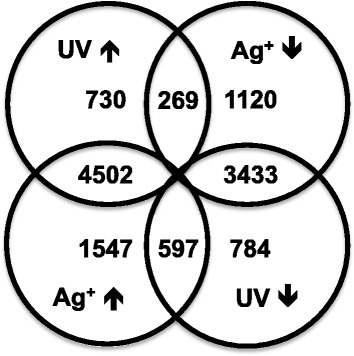
Global analysis of transcriptomics data. The set of 14,706 genes, for which RNAseq and array data could be matched, was analysed for significant (FDR P <0.05) transcriptional changes (array data) in response to UV light and AgNO_3_. A large overlap in response to the two stressors was observed.

We matched RNAseq and array data based on the comparison of array probe and transcript model sequences and omitted those probes from further analysis for which no match was found. Duplicated genes with highly homologous sequences were sometimes indistinguishable on array level (e.g. *TsCYP79B2*, see below). Here, the more highly abundant transcript from the RNAseq analysis was chosen for the matched dataset. Log_2_ fold-change values based on RNAseq and array analyses were correlated (r = 0.66 for UV light, r = 0.65 for AgNO_3_). For further analysis, we worked with a set of 14,706 genes, for which both array and RNAseq data are available (Additional file 6: Table S3). Correlations of log_2_ fold-change values in response to UV and AgNO_3_ treatment obtained by microarray hybridization are shown in Figure 2. For a large proportion of these transcripts (88%), significant changes in abundance were detected in response to UV or AgNO_3_ treatment (Figure 2). 4502 (31%) transcripts were upregulated, 3433 (23%) downregulated in response to both treatments, indicating substantial overlap in metabolic and regulatory responses.

**Figure 3:**
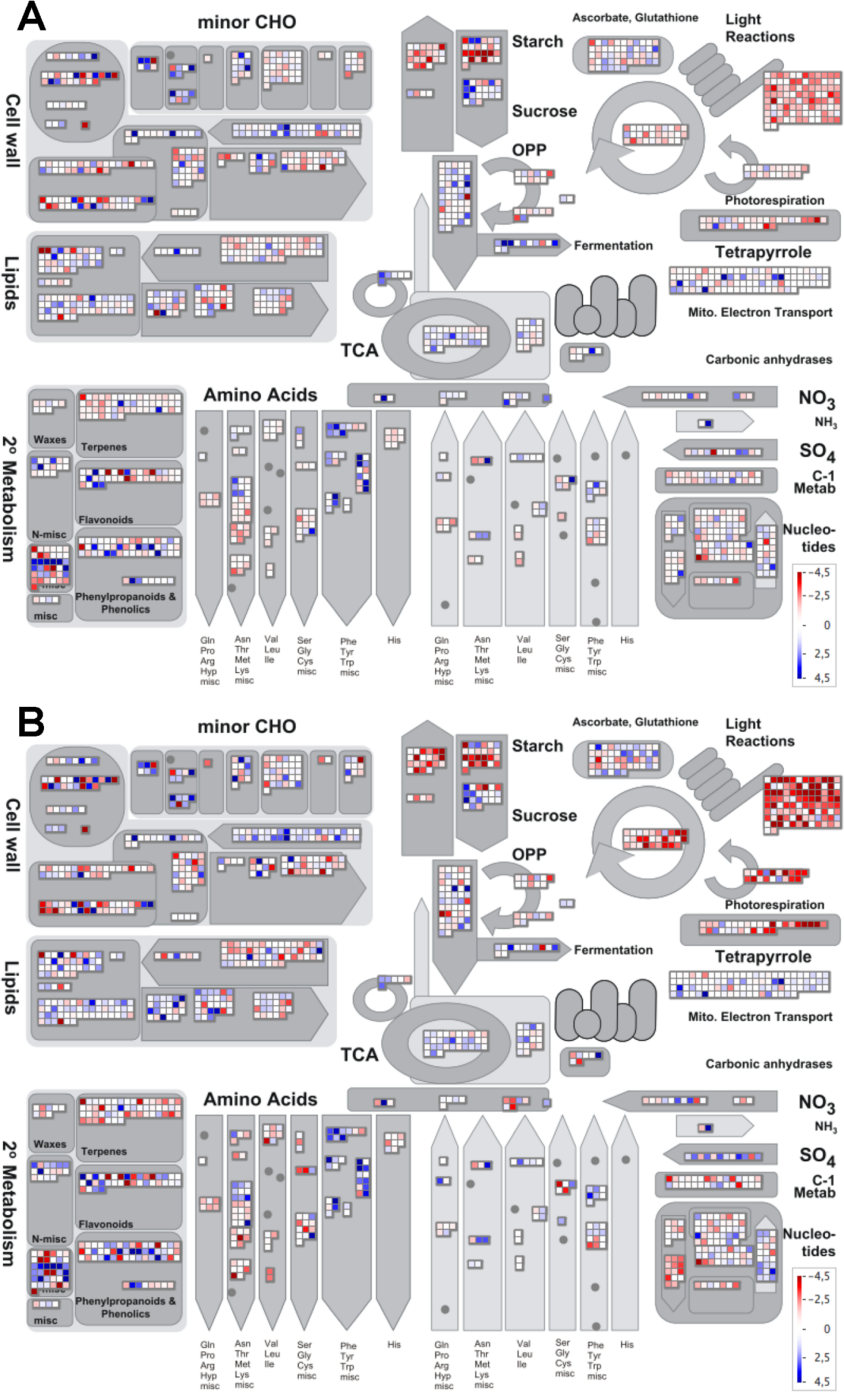
Mapman visualisation of transcript abundance changes for metabolic genes. Metabolism overview for microarray data. **A**: UV versus not induced (n.i.). **B**: AgNO_3_ versus n.i.. Red indicates downregulated, blue upregulated genes. The colour code indicates log_2_-fold changes in expression.

Figure 3 shows a Mapman [20] representation of log_2_-fold transcriptional changes, in response to UV light (Figure 3A) and AgNO_3_ (Figure 3B), based on array data. Strongly repressed processes include photosynthesis and starch synthesis. The tricarboxylic acid cycle, providing precursors of aromatic amino acid and the biosynthesis of cell wall precursors are induced on the level of transcript abundance, consistent with plant defence reactions.

### Transcriptional changes induced upon both UV and heavy metal stress

Transcripts that were strongly and consistently upregulated in response to both UV light and AgNO_3_ include a number of genes that encode enzymes involved in biosynthesis or modification of hormones and signalling compounds. This indicated that reprogramming the hormone balance is one of the key elements in the adaptation of *Eutrema* to high dosages of UV light or heavy metals. Genes upregulated most strongly in response to both stressors include *EsSOT12* and, based on NGS data, *EsST2a/EsSOT1* (Additional file 3: Table S1 and Additional file 6: Table S3). The corresponding *Arabidopsis* orthologs encode a sulfotransferase, which sulphonates salicylic acid, thereby positively regulating salicylic acid accumulation [21], and a sulfotransferase, which sulphonates hydroxyjasmonic acid [22]. *SOT12* is also strongly induced in *A. thaliana* seedlings in response to UVB light [23]. Furthermore, we observed that genes encoding *Eutrema* orthologs of 1-amino-cyclopropane-1-carboxylate synthase 2 (ethylene biosynthesis) and cis-zeatin O-β-D-glucosyltransferase (UGT85A1, cytokinin metabolism) [24] were highly upregulated in response to both UV light and AgNO_3_. Other induced processes are senescence and regulation of cell death. Here, examples of highly upregulated genes include the *Eutrema* orthologs of *AtDLAH* [25] and *AtBAP2*, an inhibitor of programmed cell death [26].

We observed significant transcriptional reprogramming of phenylpropanoid metabolism. Genes of the core phenylpropanoid biosynthetic pathway, i.e. *E. salsugineum* orthologs putatively encoding phenylalanine ammonia-lyase 1 and 2, cinnamate-4-hydroxylase, cinnamoyl CoA reductase, and cinnamyl alcohol dehydrogenase were upregulated in response to UV and AgNO_3_. The *E. salsugineum* ortholog of *TT4*, encoding naringenine chalcone synthase, was strongly downregulated. Interestingly, in *Arabidopsis* strong *TT4* upregulation was observed in response to UV light [27]. Whether this is due to experimental differences, such as plant age or UV wavelength or reflects a species-specific difference in adaptation with respect to the phenylpropanoids that are synthesized remains to be investigated. Further, fundamental changes in the transcript abundance of genes encoding enzymes involved in the biosynthesis of defence-related secondary metabolites were observed, which are discussed in detail below.

A number of genes have been functionally associated with the halophytic lifestyle of *E. salsugineum*. These include the sodium transporter *EsHKT1* [28] and *EsERF1* [29], which are also strongly and significantly upregulated under both AgNO_3_ and UV treatment (Additional file 6: Table S3). *Arabidopsis* ERF1 is an integrator of different abiotic and biotic stress responses [30]. For other genes associated with salt tolerance, such as *SOS1* and iron superoxide dismutase this was not observed [31]. We have surveyed transcriptional changes in response to AgNO_3_ and UV in *E. salsugineum* for similarity to changes in response to drought or cold [32]. There was a clear overlap among downregulated genes, which are mainly related to photosynthesis. A common pattern among upregulated genes was not observed (Additional file 7: Figure S4A). Apparently, the responses of *E. salsugineum* to drought/cold and to UV/heavy metal stresses differ substantially.

The effect of silver treatment on the *Arabidopsis* transcriptome was investigated previously by Kaveh and coworkers [33]. The number of significantly upregulated genes was much lower than in our work on *Eutrema*, probably due to differences in the experimental setup. Only for a few genes, the corresponding orthologs were identified in both studies, including the orthologs of the β-glucosidase genes 18 and 46.

Recently, genes were identified in *A. thaliana* which are upregulated in response to both *B. cinerea* infection and oxidative stress [34]. For 115 out of these 175 transcripts, corresponding *E. salsuginea* orthologs were identified here. Strikingly, for a large fraction of these genes (76; 66%), including e.g. *EsCYP79B3* and *EsCYP83B1* (see below), we observed upregulation by both UV and AgNO_3_ treatments (Additional file 7: Figure S4B). Possibly, all these processes lead to the generation of reactive oxygen species, inducing transcriptional reactions that are largely conserved between *Arabidopsis* and *Eutrema*.

### Tryptophan biosynthetic genes

In Brassicaceae, tryptophan is a precursor of indole glucosinolates and indolic phytoalexins [4], which constitute the major tryptophan sinks. As cellular tryptophan concentrations are low in *Arabidopsis* leaves, tryptophan biosynthesis is strongly coregulated with the biosynthesis of camalexin [35,36].

Here, we observed significant and strong increases in transcript levels associated with the tryptophan biosynthetic pathway in response to UV light and AgNO_3_ (Table 2). This includes genes encoding tryptophan synthase β (TSB) type 1 isoforms, while the ortholog of *TSBtype2*, of which the biological function is unknown [37], is significantly downregulated in response to UV light.

**Table 2:** **Analysis of transcript abundance changes of genes associated with the biosynthesis of defence-related metabolites**

**Transcript ID**	**Best Ath hit**	**Gene symbol**	**Annotation**	**UV**	**Ag** ^**+**^	**Fold change log2 (UV/n.i.)**	**FDR-p-value test**	**Fold change log2 (Ag/n.i.)**	**FDR-p-value test**	**RNAseq Unique reads**		
										**n.i.**	**UV**	**AgNO3**
**Tryptophan biosynthesis**												
Thhalv10013041m	AT5G05730.1	ASA1,TRP5,WEI2	anthranilate synthase alpha subunit 1	up	up	4,83	0,000	5,31	0,000	431	20815	28637
Thhalv10010558m	AT3G54640.1	TRP3,TSA1	tryptophan synthase alpha chain	up	up	4,34	0,000	3,76	0,000	264	9972	9692
Thhalv10013439m	AT4G27070.1	TSB2	tryptophan synthase beta-subunit 2	up	up	4,07	0,000	3,13	0,000	710	3153	2355
Thhalv10025097m	AT4G27070.1	TSB2	tryptophan synthase beta-subunit 2	up	up	3,37	0,000	2,47	0,000	2187	7481	5464
Thhalv10013857m	AT5G17990.1	PAT1,TRP1	tryptophan biosynthesis 1	up	up	2,26	0,000	3,26	0,000	22	207	268
Thhalv10014630m	AT4G27070.1	TSB2	tryptophan synthase beta-subunit 2	up	up	1,67	0,002	1,11	0,002	2	35	17
Thhalv10002557m	AT2G04400.1	IGPS	indole-3-glycerol phosphate synthase	up	up	1,16	0,000	1,40	0,000	684	6529	8538
Thhalv10016377m	AT2G29690.1	ASA2	anthranilate synthase 2		down	−0,20	0,356	−0,43	0,004	482	436	449
Thhalv10027732m	AT5G38530.1	TSBtype2	tryptophan synthase beta type 2	down		−1,47	0,000	−1,40	0,000	876	407	1012
**Biosynthesis of aliphatic glucosinolates**												
Thhalv10023453m	AT1G62570.1	FMO GS-OX4	glucosinolate S-oxygenase 4	up	up	4,12	0,001	3,92	0,001	243	8147	7570
Thhalv10007582m	AT1G12140.1	FMO GS-OX5	glucosinolate S-oxygenase 5	up	up	2,12	0,000	1,48	0,000	604	1102	1077
Thhalv10018813m	AT1G74090.1	ATST5B,SOT18	desulfo-glucosinolate sulfotransf. 18			2,12	0,000	1,75	0,000	1133	461	175
Thhalv10007073m	AT1G18500.1	IPMS1,MAML-4	methylthioalkylmalate synthase-like 4			0,31	0,084	0,32	0,165	1695	1815	2672
Thhalv10004037m	AT5G23010.1	IMS3,MAM1	methylthioalkylmalate synthase 1		down	−0,60	0,055	−2,92	0,000	51	1	4
Thhalv10017125m	AT2G43100.1	ATLEUD1,IPMI2	isopropylmalate isomerase 2	down		−0,97	0,002	−1,22	0,003	655	462	942
Thhalv10013695m	AT5G14200.1	IMD1	isopropylmalate dehydrogenase 1	down	down	−1,97	0,000	−1,91	0,000	1667	266	779
Thhalv10028851m	AT4G12030.2	BASS5,BAT5	bile acid transporter 5	down	down	−2,05	0,007	−4,56	0,000	11	3	2
Thhalv10024982m	AT4G13770.1	CYP83A1,REF2	cytochrome P450 83A1	down	down	−2,79	0,000	−3,97	0,000	20919	1569	2903
Thhalv10007301m	AT1G16410.1	CYP79F1	cytochrome P450 79 F1	down	down	−2,92	0,005	−5,72	0,000	15712	1101	2740
Thhalv10013952m	AT5G07690.1	MYB29	myb domain protein 29	down	down	−3,22	0,002	−4,35	0,000	3421	380	61
Thhalv10004406m	AT5G61420.2	MYB28,HAG1	myb domain protein 28	down	down	−5,60	0,000	−6,10	0,001	427	10	10
**Indole and general glucosinolate biosynthesis**												
Thhalv10007957m	AT1G21100.1	IGMT1	O-methyltransferase family protein	up	up	5,97	0,000	4,54	0,001	165	10234	6500
Thhalv10000114m	AT2G22330.1	CYP79B3	cytochrome P450 79B3	up	up	5,92	0,000	5,05	0,000	16	7685	4205
Thhalv10008152m	AT1G18570.1	AtMYB51,HIG1	myb domain protein 51	up	up	5,68	0,000	4,31	0,000	164	13093	7845
Thhalv10024861m	AT4G39950.1	CYP79B2	cytochrome P450 79B2	up	up	5,06	0,000	5,38	0,000	254	24763	30609
Thhalv10007964m	AT1G21120.1	IGMT2	O-methyltransferase family protein	up	up	4,93	0,000	2,70	0,001	434	9009	9298
Thhalv10018795m	AT1G74100.1	ATST5A,SOT16	sulfotransferase 16	up	up	4,54	0,000	4,45	0,000	665	26889	29842
Thhalv10024979m	AT4G37410.1	CYP81F4	cytochrome P450 81 F4	up	up	4,52	0,000	5,27	0,000	300	21717	48198
Thhalv10008073m	AT1G18590.1	ATST5C,SOT17	sulfotransferase 17	up	up	4,07	0,000	2,59	0,002	877	8651	4339
Thhalv10026067m	AT4G30530.1	GGP1	gammaglutamyl peptidase 1	up	up	3,31	0,000	3,55	0,000	5315	66528	98213
Thhalv10001994m	AT2G20610.1	SUR1,ALF1,RTY1	superroot1	up	up	3,22	0,000	2,48	0,000	14	28	23
Thhalv10018739m	AT1G76790.1	IGMT5	O-methyltransferase family protein	up	up	2,63	0,001	3,03	0,000	1747	42057	94252
Thhalv10007574m	AT1G24100.1	UGT74B1	UDP-glucosyl transferase 74B1	up	up	2,44	0,000	2,15	0,000	1062	6408	5926
Thhalv10024981m	AT4G37430.1	CYP81F1	cytochrome P450 81 F1		up	1,99	0,002	4,94	0,000	131	84	393
Thhalv10004064m	AT4G31500.1	CYP83B1,SUR2	cytochrome P450 83B1	up	up	1,70	0,001	2,06	0,000	2841	51916	96397
Thhalv10027443m	AT4G37400.1	CYP81F3	cytochrome P450 81 F3		up	−3,97	0,000	1,14	0,043	5	9	544
**Phenylpropanoid biosynthesis**												
Thhalv10025563m	AT4G34230.1	CAD5	cinnamyl alcohol dehydrogenase 5	up	up	4,90	0,000	4,92	0,000	590	14915	11374
Thhalv10016314m	AT2G37040.1	PAL1	PHE ammonia lyase 1	up	up	4,54	0,000	4,32	0,000	5639	43919	53026
Thhalv10010153m	AT3G53260.1	ATPAL2,PAL2	PHE ammonia lyase 2	up	up	4,47	0,000	4,08	0,000	5516	24942	29448
Thhalv10016545m	AT2G30490.1	C4H,CYP73A5	cinnamate-4-hydroxylase	up	up	4,47	0,000	3,92	0,000	3465	40914	24286
Thhalv10018849m	AT1G80820.1	CCR2	cinnamoyl coa reductase	up	up	4,38	0,000	3,95	0,000	9	2796	6118
Thhalv10016544m	AT2G30490.1	CYP73A5,REF3	cinnamate-4-hydroxylase	up	up	4,34	0,000	4,28	0,000	200	5351	6505
Thhalv10020406m	AT3G21230.1	4CL5	4-coumarate:CoA ligase 5	up	up	2,86	0,000	1,30	0,000	160	961	882
Thhalv10001440m	AT2G43820.1	SGT1,UGT74F2	UDP-glucosyltransferase 74 F2	up	up	2,58	0,001	4,45	0,000	98	1100	1193
Thhalv10004662m	AT5G08640.1	FLS1	flavonol synthase 1	up	up	2,41	0,003	0,80	0,026	52	416	498
Thhalv10016538m	AT2G40890.1	CYP98A3	cytochrome P450, 98A3			2,32	0,001	2,10	0,000	1482	847	699
Thhalv10011357m	AT1G51680.3	4CL1	4-coumarate:CoA ligase 1	up	up	1,85	0,002	1,35	0,000	875	3071	2269
Thhalv10010658m	AT3G55120.1	A11,CFI,TT5	Chalcone-flavanone isomerase	up	up	1,76	0,001	2,04	0,000	112	352	573
Thhalv10024928m	AT4G36220.1	CYP84A1,FAH1	ferulic acid 5-hydroxylase 1	up	up	1,51	0,002	1,83	0,000	5064	5573	8972
Thhalv10026028m	AT4G34050.1	CCoAOMT1	SAM-dependent methyltransferase	up		0,99	0,010	0,04	0,779	851	3093	3791
Thhalv10000324m	AT3G21230.1	4CL5	4-coumarate:CoA ligase 5		up	0,85	0,021	0,75	0,001	28	16	240
Thhalv10008111m	AT1G15950.1	CCR1	cinnamoyl coa reductase 1	up		0,76	0,008	0,15	0,447	423	586	375
Thhalv10000513m	AT3G21230.1	4CL5	4-coumarate:CoA ligase 5			0,27	0,533	−0,03	0,957	21	17	339
Thhalv10018769m	AT1G72680.1	CAD1	cinnamyl-alcohol dehydrogenase		down	−0,15	0,461	−0,23	0,050	859	635	666
Thhalv10022462m	AT1G65060.1	4CL3	4-coumarate:CoA ligase 3		down	−0,25	0,141	−0,55	0,004	113	23	15
Thhalv10020439m	AT3G21230.1	4CL5	4-coumarate:CoA ligase 5	down	down	−0,83	0,015	−0,93	0,004	910	214	484
Thhalv10027317m	AT4G36220.1	CYP84A1,FAH1	ferulic acid 5-hydroxylase 1			−0,98	0,338	−1,38	0,170	1	1	3
Thhalv10013289m	AT5G07990.1	CYP75B1,TT7	cytochrome P450, 75B1			−1,26	0,080	−1,16	0,084	4	2	33
Thhalv10004668m	AT5G08640.1	ATFLS1,FLS,FLS1	flavonol synthase 1	down	down	−1,43	0,005	−2,38	0,002	137	17	41
Thhalv10005442m	AT1G43620.1	TT15,UGT80B1	UDP-Glycosyltransferase 80B1	down	up	−1,72	0,000	0,34	0,008	1061	827	4174
Thhalv10014054m	AT5G08640.1	FLS1	flavonol synthase 1	down		−2,67	0,000	−2,92	0,001	17	11	19
Thhalv10013745m	AT5G13930.1	CHS,TT4	Chalcone and stilbene synth. Fam.	down	down	−4,04	0,000	−3,32	0,005	214	11	200

### Glucosinolate biosynthesis and modification

Members of the order Brassicales synthesize glucosinolates from non-polar amino acids as major defence compounds against herbivores and pathogens. In *Arabidopsis thaliana,* almost exclusively methionine-derived aliphatic and tryptophan-derived indole glucosinolates are found. Their biosynthetic pathways are known in great detail [38]. In *Eutrema salsugineum* Shandong, the short chain aliphatic allyl-2-phenylethyl-, 3-methylsulfinylpropyl-, and 3-methylthiopropylglucosinolate, the very-long-chain aliphatic 10-methylsulfinyldecylglucosinolate, as well as 3-indolylmethyl- and 1-methoxy-3-indolylmethylglucosinolate were identified as major compounds [11] (*E. salsugineum* denoted in this publication as *T. halophila*). According to labelling experiments, 1-methoxy-3-indolylmethylglucosinolate is likely to be a biosynthetic intermediate of the phytoalexins 1-methoxybrassinin and wasalexin A and B [18].

For all defined steps of the core aliphatic and indole glucosinolate biosynthetic pathways, putative orthologs of the genes encoding the corresponding enzymes were found in *Eutrema salsugineum*, based on homology and synteny to *A. thaliana*. Some additional duplication events or losses of tandem copies were detected. In contrast to the tandem duplicates *CYP79F1* and *CYP79F2* in *A. thaliana*, only one copy, designated as *EsCYP79F1* was found in *E. salsugineum*, suggesting that this single gene is essential for the biosynthesis of aliphatic glucosinolates. A putative *CYP79A2* [39] ortholog was found, which is expressed at very low levels (0, 0, and 1 reads in n.i., UV, and AgNO_3_ samples, respectively) consistent with the apparent absence of phenylalanine-derived glucosinolates [11]. *E. salsugineum* contains three *CYP79B* genes due to a recent duplication of *CYP79B2* leading to two transcripts hybridizing to the same array probe and generating proteins with 98.6% identity of their amino acid sequences. These two duplicates strongly differ in expression level based on RNAseq data (254, 24763 and 30609, versus 0, 3, and 5 reads in n.i., UV, and AgNO_3_ samples, respectively).

In response to UV light and AgNO_3_, the core genes of indole glucosinolate biosynthesis are strongly upregulated, consistent with the proposed role of 1-methoxy-3-indolylmethylglucosinolate as precursor of the characteristic *Eutrema* phytoalexins (Table 2). Also, the ortholog of *MYB51*/*HIG1*, encoding a master regulator of indole glucosinolate biosynthesis in *Arabidopsis* [40], is consistently induced. Strikingly, in response to these stressors, transcripts encoding indole glucosinolate biosynthetic genes, such as *EsCYP83B1* and *EsGGP1* are among the most highly abundant, according to our RNAseq data, indicating an important metabolic response.

In *Arabidopsis*, a time course experiment has been performed for UV response [41]. We surveyed these data for the responses of orthologs of *E. salsugineum* genes we analysed by RT-qPCR (Figure 1). Moderate upregulation with respect to 0 h, peaking at 3 h for *AtASA1* (5.0-fold) and *AtPEN2* (2.0-fold), and at 6 h for *AtIGMT5* (3.6-fold) and *AtBGLU18* (3.3-fold) was observed. More generally, we surveyed these data for core indole glucosinolate biosynthetic genes and again observed only modest transcript induction 6 h after UV treatment (less than 5-fold upregulation of *CYP83B1*, *SUR1*, *GGP1*, *SOT16* and *UGT74B1*). In contrast, the camalexin biosynthetic genes *CYP71B15* and *CYP71A13* were induced approximately 121-fold and 66-fold, respectively [41]. These differential responses are consistent with the proposed phytoalexin biosynthetic pathways in the two species.

In *Arabidopsis*, unmodified indole glucosinolate is methoxylated in response to pathogen infection, involving members of the CYP81F family and indole glucosinolate methyl transferases (IGMTs) [42]. *E. salsugineum* contains five *CYP81F* members, due to an additional gene copy in the *CYP81F1/3/4* cluster. For three of these genes, microarray and RNAseq data were obtained and matched. Based on its expression pattern, *EsCYP81F4* (Thhalv10024979m) is a candidate gene for catalysing N-hydroxylation of 3-indolylmethylglucosinolate in the biosynthesis of *Eutrema* phytoalexins. *EsCYP81F3* (Thhalv10027443m) was induced by AgNO_3_ but not by UV light. Also, *EsIGMT5,* highly expressed in response to stress treatment (Table 2, Figure 1), is a candidate for involvement in the biosynthesis of N-methoxylated indolic compounds.

In response to pathogen infection, in *Arabidopsis* indole glucosinolates are degraded to bioactive compounds by the β-glucosidase PEN2 (BGLU26) [43,44]. We hypothesize that β-glucosidases are also involved in the biosynthesis of *Eutrema* phytoalexins. A number of β-glucosidase-encoding genes were significantly upregulated in response to AgNO_3_ and UV challenge (Table 3), including *EsPEN2* (Thhalv10001354m), *EsBGLU18-1* (Thhalv10011384m)*,* and *EsBGLU18-2* (Thhalv10011385m). The time course of induction of these genes was monitored by quantitative RT-PCR and strong induction responses to AgNO_3_ and UV treatment were confirmed (Figure 1). In conclusion, the *Eutrema* orthologs of *PEN2* (*BGLU26*) and *BGLU18* are candidates for an involvement in phytoalexin biosynthesis.

**Table 3:** **Analysis of transcript abundance changes of genes encoding β-glucosidases**

**Transcript ID**	**Best Ath hit**	**Gene symbol**	**UV**	**Ag** ^**+**^	**Fold change log2 (UV/n.i.)**	**FDR-p-value test**	**Fold change log2 (Ag/n.i.)**	**FDR-p-value test**	**RNAseq Unique reads**		
									**n.i.**	**UV**	**AgNO3**
Thhalv10006515m	AT4G27830.1	BGLU10	up		1,80	0,001	−1,56	0,000	3	30	6
Thhalv10006510m	AT4G27830.1	BGLU10	up		1,42	0,000	−1,94	0,000	40	389	105
Thhalv10005908m	AT4G27830.1	BGLU10	down		−2,08	0,000	−1,85	0,000	217	127	587
Thhalv10001447m	AT2G44450.1	BGLU15	up	up	1,14	0,003	2,08	0,000	2	20	29
Thhalv10011384m	AT1G52400.1	BGLU18	down	up	−5,32	0,000	0,47	0,000	4823	2272	195299
Thhalv10011385m	AT1G52400.1	BGLU18			−7,87	0,000	0,41	0,279	2	6	1246
Thhalv10020508m	AT3G09260.1	BGLU23,PYK10		up	0,18	0,650	1,20	0,045	19	106	861
Thhalv10020496m	AT3G03640.1	BGLU25,GLUC		up	−0,13	0,582	1,46	0,001	415	811	1634
Thhalv10001354m	AT2G44490.1	BGLU26,PEN2	up	up	1,94	0,000	1,70	0,000	2401	11117	21129
Thhalv10002501m	AT4G22100.1	BGLU3			−1,19	0,036	−0,22	0,547	2	20	542
Thhalv10004297m	AT4G22100.1	BGLU3	down	down	−1,30	0,000	−2,00	0,000	11	3	8
Thhalv10028552m	AT4G22100.1	BGLU3			−1,85	0,000	−1,19	0,000	85	89	170
Thhalv10002493m	AT4G22100.1	BGLU3	down		−2,57	0,000	−1,31	0,001	100	92	571
Thhalv10005858m	AT3G60140.1	BGLU30,SRG2		up	0,50	0,566	6,19	0,000	5	18	344
Thhalv10018387m	AT5G24550.1	BGLU32	up	up	5,96	0,000	6,62	0,000	729	31451	52650
Thhalv10002474m	AT5G26000.1	BGLU38,TGG1			−0,37	0,529	−0,38	0,598	7	0	2
Thhalv10004165m	AT5G26000.1	BGLU38,TGG1			−0,68	0,303	−0,73	0,375	11	1	2
Thhalv10003954m	AT5G26000.1	BGLU38,TGG1			−1,29	0,085	−0,88	0,219	6	0	10
Thhalv10007404m	AT1G26560.1	BGLU40	up	up	2,11	0,000	2,54	0,000	167	652	1015
Thhalv10027734m	AT5G36890.1	BGLU42	down		−1,09	0,000	0,24	0,001	773	351	731
Thhalv10020536m	AT3G18080.1	BGLU44			1,81	0,002	1,01	0,006	8	5	3
Thhalv10023411m	AT1G61820.1	BGLU46	up	up	2,81	0,005	7,02	0,000	1	157	964

In response to UV light and AgNO_3_, most genes involved in aliphatic glucosinolate biosynthesis were strongly downregulated, with the exception of the putative orthologs of flavin-containing monooxygenase (FMO) genes encoding glucosinolate S-oxygenases (Table 2), probably resulting in a metabolic shift towards indolic and oxidized aliphatic glucosinolates. Based on homology and chromosomal position Thhalv10008073m is orthologous to *AtSOT17/AtST5c* (At1g18590), encoding a sulfotransferase with a preference for aliphatic desulfoglucosinolates as substrates [45,46]. Here, we observed strong transcriptional upregulation of *EsSOT17* (Thhalv10008073m) in response to UV irradiation and AgNO_3_ treatment, similar to genes involved in indole glucosinolate biosynthesis. We speculate that in the two species the two orthologs have acquired different substrate specificities and that the *Eutrema* gene functions in indole glucosinolate biosynthesis. The two other confirmed desulfoglucosinolate sulfotransferases AtSOT18/AtST5b and AtSOT16/AtST5a have probably retained their function in aliphatic and indole glucosinolate biosynthesis, respectively.

### WRKY transcription factors

In *Arabidopsis*, WRKY transcription factors play an essential role in the regulation of phytoalexin responses. Our data show that also in *Eutrema* several *WRKY* genes are upregulated, including the orthologs of *WRKY40*, *WRKY75*, *WRKY33*, *WRKY6*, *WRKY51* and *WRKY18* (Table 4). WRKY18 and WRKY40 are central regulators of indole glucosinolate modification in response to pathogens [47]. WRKY6 is associated with both senescence- and defence-related processes [48] and WRKY75, besides its role in phosphate acquisition [49], is also linked to senescence and pathogen defence [50,51]. WRKY51 plays a role in modulation of salicylate- and jasmonate signalling in defence [52]. In summary, these transcriptional changes indicate that also in *Eutrema* WRKYs are crucial for induced metabolic defence.

**Table 4:** **Analysis of transcript abundance changes of genes encoding WRKY transcription factors**

**Transcript ID**	**Best Ath hit**	**WRKY**	**UV**	**Ag** ^**+**^	**Fold change log2 (UV/n.i.)**	**FDR-p-value test**	**Fold change log2 (Ag/n.i.)**	**FDR-p-value test**	**RNAseq Unique reads**		
									**n.i.**	**UV**	**AgNO3**
Thhalv10002516m	AT2G04880.2	1	down	down	−0,16	0,026	−0,39	0,000	170	79	89
Thhalv10012829m	AT5G56270.1	2		up	0,35	0,144	0,37	0,014	742	649	786
Thhalv10004015m	AT2G03340.1	3			−0,27	0,315	−0,02	0,923	746	866	648
Thhalv10007428m	AT1G13960.1	4	up	up	2,27	0,000	2,56	0,000	1482	1556	2016
Thhalv10023390m	AT1G62300.1	6	up	up	4,84	0,000	5,03	0,000	83	7722	9852
Thhalv10025630m	AT4G24240.1	7			−0,25	0,122	−0,21	0,241	384	161	260
Thhalv10025646m	AT4G31550.1	11	up	up	1,93	0,001	3,53	0,000	120	700	3144
Thhalv10000270m	AT2G23320.1	15	up	up	4,43	0,000	3,38	0,000	757	4909	2951
Thhalv10001165m	AT5G45050.2	16			−0,22	0,641	0,64	0,116	5963	1782	5082
Thhalv10000242m	AT2G24570.1	17	up	up	1,64	0,001	2,40	0,000	300	1089	2481
Thhalv10025785m	AT4G31800.1	18	up	up	2,15	0,002	3,61	0,000	173	1582	2793
Thhalv10024810m	AT4G26640.2	20		down	−0,17	0,133	−0,38	0,029	646	333	300
Thhalv10013115m	AT4G26640.2	20	down		−1,46	0,035	0,40	0,637	367	124	210
Thhalv10016852m	AT2G30590.1	21	up		0,69	0,003	0,40	0,001	8	12	7
Thhalv10028843m	AT4G01250.1	22	up	up	2,88	0,000	4,37	0,000	7	50	211
Thhalv10016764m	AT2G30250.1	25	up	up	1,79	0,001	2,06	0,000	239	1182	1844
Thhalv10017017m	AT2G30250.1	25	up	up	1,55	0,010	2,22	0,000	22	120	136
Thhalv10013872m	AT5G52830.1	27		down	0,72	0,036	−1,29	0,005	48	27	10
Thhalv10025799m	AT4G23550.1	29			1,71	0,009	−0,42	0,428	18	16	23
Thhalv10025126m	AT4G30935.1	32		down	−0,17	0,244	−0,33	0,049	1203	391	285
Thhalv10016542m	AT2G38470.1	33	up	up	5,31	0,000	4,37	0,000	325	15433	11838
Thhalv10021115m	AT3G04670.1	39		up	1,14	0,001	1,98	0,000	169	143	195
Thhalv10018925m	AT1G80840.1	40	up	up	6,44	0,000	4,50	0,000	23	7398	7919
Thhalv10028794m	AT4G11070.1	41	up	up	4,24	0,000	3,22	0,000	6	46	85
Thhalv10001568m	AT2G46400.1	46	up	up	3,15	0,000	4,01	0,000	72	925	2639
Thhalv10005029m	AT5G26170.1	50		up	0,17	0,680	1,30	0,005	41	67	93
Thhalv10004930m	AT5G64810.1	51	up	up	4,29	0,000	4,30	0,000	55	1520	1853
Thhalv10016713m	AT2G40750.1	54	up	up	1,27	0,005	2,38	0,000	407	1840	3229
Thhalv10017926m	AT2G40740.1	55	up	up	6,27	0,000	4,45	0,000	31	241	411
Thhalv10018976m	AT1G69310.1	57			0,12	0,559	0,28	0,173	410	196	249
Thhalv10000288m	AT2G25000.1	60	down	down	−2,14	0,000	−0,70	0,002	99	37	90
Thhalv10006157m	AT3G58710.1	69	up		−0,52	0,162	1,54	0,001	34	48	194
Thhalv10006146m	AT3G56400.1	70	up	up	1,66	0,002	4,40	0,000	216	2525	12004
Thhalv10013146m	AT5G15130.1	72		up	0,30	0,450	3,54	0,000	1	1	25
Thhalv10014943m	AT5G13080.1	75	up	up	6,26	0,000	7,02	0,000	9	1076	1311

### EsWRKY33 complements camalexin deficiency in an Arabidopsis WRKY mutant

In *Arabidopsis*, WRKY33 is an essential regulator of camalexin biosynthesis and directly binds to the promoter of *CYP71B15* (*PAD3*) [19]. Accordingly, its expression is induced by Pathogen-associated molecular patterns (PAMPs) and it is important for resistance against necrotrophic fungal pathogens [53-56]. Camalexin has not been detected in *Eutrema* and it does not contain a clear ortholog of *CYP71B15*. The closest CYP71B15 homolog in *E. salsugineum* shares only 66.7% identical amino acids. Nevertheless, *EsWRKY33* is strongly upregulated upon phytoalexin inducing conditions (Figure 1; Table 4).

**Figure 4:**
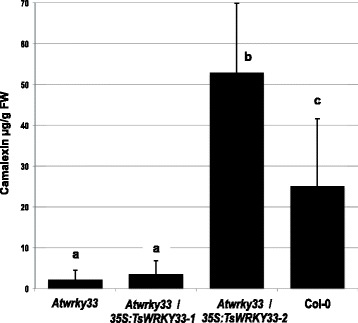
Complementation of camalexin deficiency in *Arabidopsis wrky33* knockout mutant by *EsWRKY33* expression. All plants were induced by UV light and analysed 20 h after the onset of induction. Mean and standard deviation is depicted. Different letters above the bars indicate significantly different amounts of camalexin in the respective samples, as determined by one-way ANOVA (Bonferrfoni; p < 0.05); n = 11.

We investigated whether EsWRKY33 can functionally replace AtWRKY33 as a positive regulator of camalexin biosynthesis and expressed *EsWRKY33* in the *Arabidopsis wrky33-1* mutant [54]. While in *wrky33* leaves camalexin levels were significantly reduced in relation to wild type, wild type levels were restored in the complementing line (Figure 4). This indicates that even though *Eutrema* does not synthesize camalexin, EsWRKY33 can act as a positive regulator of camalexin biosynthesis in *Arabidopsis*.

## Conclusions

In *E. salsugineum*, UV irradiation or heavy metal application resulted in substantial transcriptional reprogramming consistent with the induction of defence responses. Photosynthesis and starch synthesis were transcriptionally downregulated, while processes providing precursors for aromatic defence metabolites and cell wall compounds were transcriptionally induced. Strikingly, a shift in expression is observed from orthologs of genes for the biosynthesis of aliphatic glucosinolates, probably functioning primarily in insect defence, to orthologs of genes for the biosynthesis of indole glucosinolates, serving as precursors of phytoalexins.

WRKY33 is an essential regulator of the camalexin biosynthetic gene *CYP71B15* (*PAD3*) [19], for which there is probably no functional homolog in *E. salsugineum*, consistent with the absence of camalexin in this species [12]. Nevertheless, there is a putative *Eutrema WRKY33* ortholog, which is strongly upregulated under phytoalexin inducing conditions. *EsWRKY33* was functionally tested and shown to complement camalexin deficiency in an *Atwrky33* mutant. We hypothesize that regulatory mechanisms for phytoalexin induction are conserved among members of the Brassicaceae, while the individual chemical structures have strongly diversified.

## Methods

### Plant growth conditions and stress treatments

After 10 days (*E. salsugineum*) or two days (*A. thaliana*) of stratification at 6°C, plants were grown in a growth chamber at a 12/12 h photoperiod at a light intensity of 80 to 100 μmol m^−2^ s^−1^ at 21°C and 40% relative humidity. For stress treatment leaves were sprayed with 5 mM AgNO_3_ or placed under a UV lamp (Desaga UVVIS, λ = 254 nm, 8 W) at a distance of 20 cm and radiated for 2 h. For *Botrytis cinerea* infection a spore suspension (strain B05.10, 2 × 10^5^ spores per ml) was sprayed on the leaf surface.

### RNA isolation, cDNA preparation and RT-qPCR

RNA extraction, cDNA synthesis and RT-qPCR, performed with the SYBRGreen/Light Cycler system (Roche), has been described previously [57]. The following primers were used:

- *AtActin1*: 5′TGGAACTGGAATGGTTAAGGCTGG3′ and 5′TCTCCAGAGTCGAGCACAATACCG3′
- *AtGAPC*: 5′GCACCTTTCCGACAGCCTTG3′ and 5′ATTAGGATCGGAATCAACGG3′
- *EsActin1*: 5′TGGAACTGGAATGGTTAAGGCTGG3′ and 5′TCTCCAGAGTCGAGCACAATACCG3′
- *EsYLS8*: 5′GCGATTCTGGCTGAGGAAGA3′ and 5′CTTCCTTGCACCACGGTAGA3′
- *EsPP2AA2*: 5′TGCTGAAGATAGGCACTGGA3′ and 5′CATTGAATTTGATGTTGGGAAC3′
- *EsASA1*: 5′ATGTCTAGCGTTGGTCGTTATAGCG3′ and 5′CTTGACCACAGCCTCCTTGTACTCT3′
- *EsIGMT5*: 5′AGTGCCAAGTCGTTGATGGT3′ and 5′TTGATACCCTTGATGTTTGGA3′
- *EsBGLU18-1*: 5′AGAGGACCTTGGAGACCTTC3′ and 5′AGTTCTTCCCTCACTAACTTGGA3′
- *EsBGLU18-2*: 5′CCTACTCGTGCTCTACTGGA3′ and 5′TCCCGGCTTAAGGAAATCAGA3′
- *EsPEN2*: 5′CCAACAGGACTCAGAAACGT3′ and 5′GCAGTGACAACGAACAAGCT3′
- *EsWRKY33*: 5′TATCCATTCACAGGAACAACAGAG3′ and 5′GGATGGTTATGGCTTCCCTT3′.

Expression values of candidate genes were normalized to the geometric mean of three reference genes [58] (*EsActin1*, *EsYELLOW-LEAF-SPECIFIC GENE 8* (*EsYLS8*), and *EsPROTEIN PHOSPHATASE 2A SUBUNIT A2* (*EsPP2AA2*)). Expression level of *EsWRKY33* in the *A. thaliana* background was normalized to *AtActin1* and *AtGAPC*.

### RNAseq setup and analysis

Total RNA was isolated from three biological replicates of either control leaves or from leaves treated either with UV light for 2 h followed by 6 h recovery, leaves sprayed with 5 mM AgNO_3_ and incubated 8 h, or 48 h after infection of plants with *B. cinerea*, using the NucleoSpin® RNA II Kit (Machery-Nagel). Single-end cDNA libraries were prepared and sequenced using Illumina HiSeq 2000 technology at LGC Genomics [59] to obtain 50 bp reads. Demultiplexing was done using Illumina’s CASAVA software [60]. Reads were adapter-clipped and reads shorter than 20 bases were discarded. Read quality was assessed using FASTQC [61]. Table 1 lists the resulting number of reads used for analyses.

CLC Genomics workbench Version 6.5.1 [62] was used for RNAseq analysis including mapping to the *Eutrema* reference transcriptome [9] using default settings, allowing for at most two mismatches and a maximum of 10 transcript hits per read and generation of RPKM value statistic. Differential gene expression was detected using Fisher exact tests based on mapped read counts and with FDR-based correction for multiple testing errors [63]. Fold changes were computed using RPKM-values.

### Microarray setup and statistical analysis

For each treatment, four biological replicates were investigated, generated from pooled tissues of 4 plants. Total RNA was extracted with NucleoSpin® RNA II Kit (Machery-Nagel). After DNase treatment, concentration and quality of extracted RNA was measured photometrically and with a Bioanalyzer (Agilent Technologies, Santa Clara, CA). Samples were hybridized to Agilent 8 × 60 k microarrays by OakLabs GmbH [64]. The arrays contain 42,562 oligonucleotide probes and are based on the recently developed Agilent 4 × 44 k *Eutrema* array [9].

Raw hybridization signals were quantile-normalized and log-base-2 transformed. Differential gene expression was assessed using ANOVA across all conditions and repeats and t-test statistic for pairwise comparisons with FDR-multiple testing correction [63]. Differential gene expression was mapped to metabolic pathways using the MAPMAN software [20].

### RNAseq and microarray data match, functional annotation/candidate orthologs in *Arabidopsis thaliana*

Mapping of array probe identifiers to reference transcriptome identifiers was based on sequence matches using BLASTN with an E-value cutoff of 1E-05. Candidate ortholog genes in *Arabidopsis thaliana* were identified as best sequence identity hits using BLASTN with the same cutoff. The set of representative *Arabidopsis* transcripts available from TAIR10 [65] was used.

### Generation of WRKY33 complementation lines

*EsWRKY33* (Thhalv10016542m) coding sequence was amplified from cDNA (*E. salsugineum* leaves, 5 h after UV treatment) using the primer pair 5′GGCTTAAUATGGCTGCTTCTTCTCTTC3′ and 3′GGTTTAAUTCACGACAAAAACGAATCAAA5′ and cloned into pCAMBIA330035Su via USER technology [66]. After confirmation of the correct cDNA sequence, *Agrobacterium*-mediated transformation of *Arabidopsis wrky33-1* knockout mutant (SALK_006603; [54]) was performed via floral-dipping, and successful transformants were confirmed by BASTA resistance of the seedlings and by PCR analysis. Primary transformants were analysed for *EsWRKY33* expression by RT-qPCR. One low (#1, 0.48 ± 0.26 fg/fg *AtActin1*, 0.11 ± 0.09 fg/fg *GAPC*) and one high (#2, 16.2 ± 7.8 fg/fg *AtActin1*, 2.8 ± 1.7 fg/fg *GAPC*) expression line was selected for phenotype analysis.

### Metabolite analysis

Camalexin formation was induced in six-week old *A. thaliana* plants by treatment with UV (see above). Camalexin was isolated 20 h after the onset of induction and quantified applying HPLC with fluorescence detection as described [67]. For monitoring wasalexin A formation, *E. salsugineum* leaves were treated with either UV light for 2 h followed by 22 h incubation, sprayed with 5 mM AgNO_3_ and incubated 24 h, or sprayed with *B. cinerea* spore suspension and incubated 48 h. Plant material was frozen in liquid nitrogen. Leaves were ground and 900 μl methanol were added. The samples were incubated at room temperature for 30 min under agitation, centrifuged for 15 min at 14,000 rpm and the supernatant was transferred to a new tube. To increase the yield of metabolites the extraction was repeated once and supernatants were combined. The solvent was evaporated completely (SA-Speed Concentrator, H.Saur Laborbedarf) and metabolites were dissolved in 400 μl 100% methanol. Quantification of Wasalexin A was done via reverse-phase HPLC (Göhler Multohigh100 RP-18, 5 μm, 250mmx4mm; flow rate: 1 ml/min; solvents: acetonitrile and 0.3% formic acid in H_2_O; 20% acetonitrile for 2 min, followed by a 17 min linear gradient to 70% acetonitrile and then 3 min to 100% acetonitrile). The peaks at 20.2 min and 21.5 min (OD_max_: 362 nm) were identified as Wasalexin B and Wasalexin A, respectively by comparison with authentic standard with respect to retention time and UV spectrum.

### Availability of supporting data

All curated supporting data are included as additional files. The raw RNAseq data have been deposited in the National Center for Biotechnology Information (NCBI) Sequence Read Archive (SRA) database under the accession number SRP048695. Microarray data was deposited at Gene Expression Omnibus (GEO) database under the accession numbers GSM1530883 to GSM1530894 (platform accession GPL19319).

## Additional files

Additional file 1: Figure S1. Induction of wasalexin in response to UV light, AgNO_3_ and *B. cinerea.* Leaf extracts (24 h after onset of induction by UV or AgNO_3_ or 48 h after fungal infection) and authentic standard were analysed by HPLC (see Methods section). Representative chromatograms at 363 nm are shown. Additional file 2: Figure S2. Venn diagram representing uniquely mapped transcripts identified in RNAseq of the four cDNA libraries. Additional file 3: Table S1. Complete dataset for RNAseq analysis. Additional file 4: Table S2. Complete dataset for microarray analysis. Additional file 5: Figure S3. Correlation of log-fold changes between RNAseq and array data after matching. A: UV versus not induced (n.i.). B: AgNO_3_ versus n.i. Additional file 6: Table S3. Complete dataset for 14706 genes, for which both array and RNAseq data is available. Additional file 7: Figure S4. UV- and AgNO_3_-responsiveness of genes differentially regulated in other studies. A: 81 drought and 59 cold regulated *E. salsugineum* genes (Wong et al. [32]) B: Identified putative orthologs of A. thaliana genes upregulated in response to both *B. cinerea* and oxidative stress (115 identified, 7 unchanged in response to UV or Ag^+^).

## Abbreviations

ANOVA: Analysis of variance
FDR: False discovery rate
JGI: Joint Genome Institute
RPKM: Reads per kilobase of transcript per million reads mapped
RT-qPCR: Reverse transcription quantitative polymerase chain reaction
