# Supplementary material for: Substantial reprogramming of the Eutrema salsugineum (Thellungiella salsuginea) transcriptome in response to UV and silver nitrate challenge
Source: BMC Plant Biol. 2015 Jun 12;15:137. doi: 10.1186/s12870-015-0506-5 (PMC4464140; doi:10.1186/s12870-015-0506-5)
Supplement: Additional file 1: Figure S1. — Induction of wasalexin in response to UV light, AgNO3 and B. cinerea. Leaf extracts (24 h after onset of induction by UV or AgNO3 or 48 h after fungal infection) and authentic standard were analysed by HPLC (see Methods section). Representative chromatograms at 363 nm are shown. [file 12870_2015_506_MOESM1_ESM.docx]

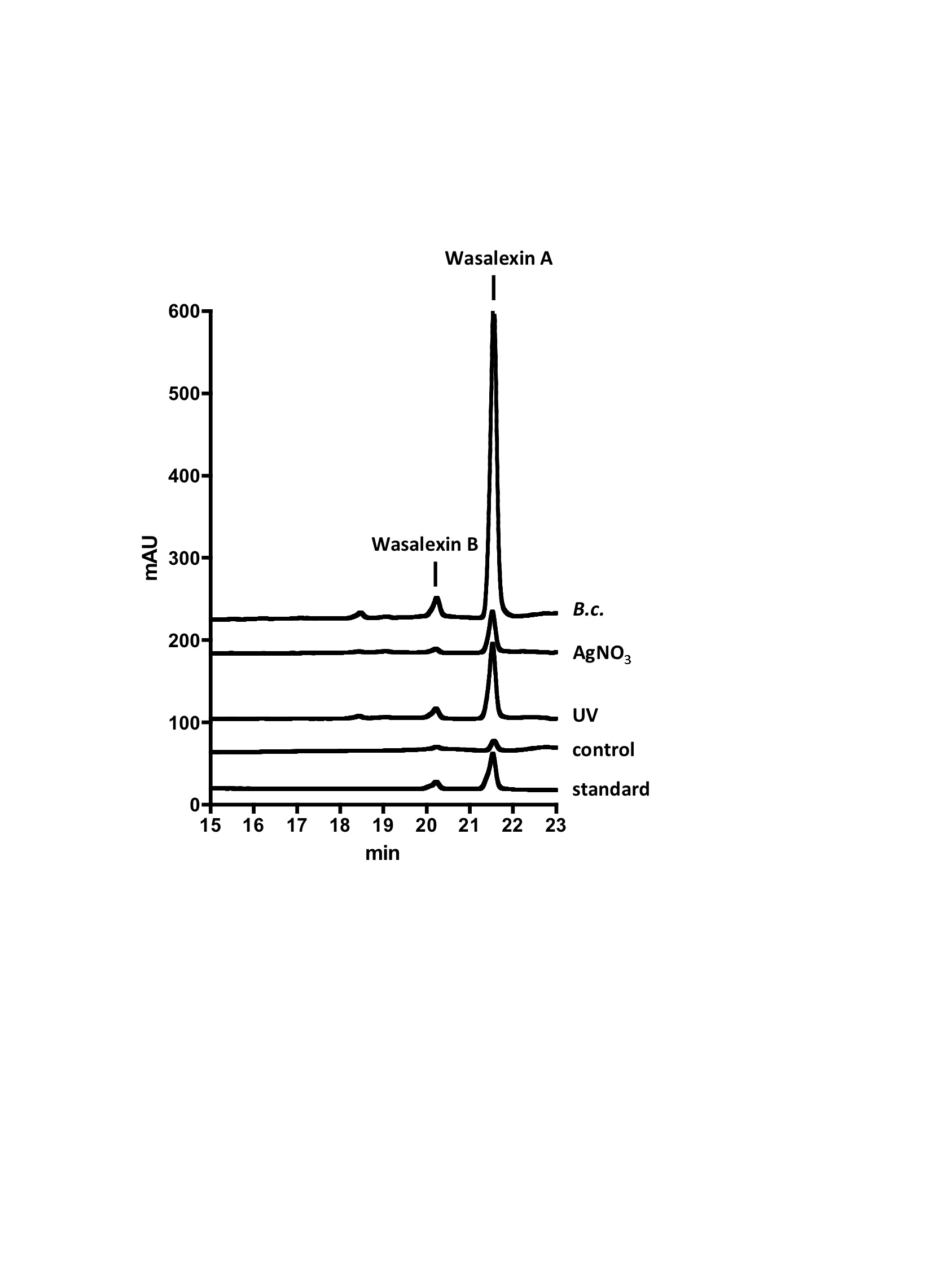


**Supplemental Fig. 1: Induction of wasalexin in response to UV, AgNO_3_ and *B. cinerea* (*B.c.*)*.*** Leaf extracts (24 h after onset of induction by UV or AgNO_3_ or 48 h after fungal infection) and authentic standard were analysed by HPLC (see methods section). Representative chromatograms at 363 nm are shown.
