# Supplementary figures and images for: Substantial reprogramming of the Eutrema salsugineum (Thellungiella salsuginea) transcriptome in response to UV and silver nitrate challenge
Source: BMC Plant Biol. 2015 Jun 12;15:137. doi: 10.1186/s12870-015-0506-5 (PMC4464140; doi:10.1186/s12870-015-0506-5)

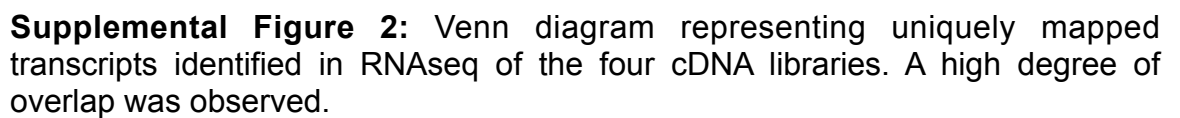

Supplement: Additional file 2: Figure S2. — Venn diagram representing uniquely mapped transcripts identified in RNAseq of the four cDNA libraries. [file 12870_2015_506_MOESM2_ESM.pdf]
