# Supplementary material for: Substantial reprogramming of the Eutrema salsugineum (Thellungiella salsuginea) transcriptome in response to UV and silver nitrate challenge
Source: BMC Plant Biol. 2015 Jun 12;15:137. doi: 10.1186/s12870-015-0506-5 (PMC4464140; doi:10.1186/s12870-015-0506-5)
Supplement: Additional file 5: Figure S3. — Correlation of log-fold changes between RNAseq and array data after matching. A: UV versus not induced (n.i.). B: AgNO3 versus n.i. [file 12870_2015_506_MOESM5_ESM.pdf]

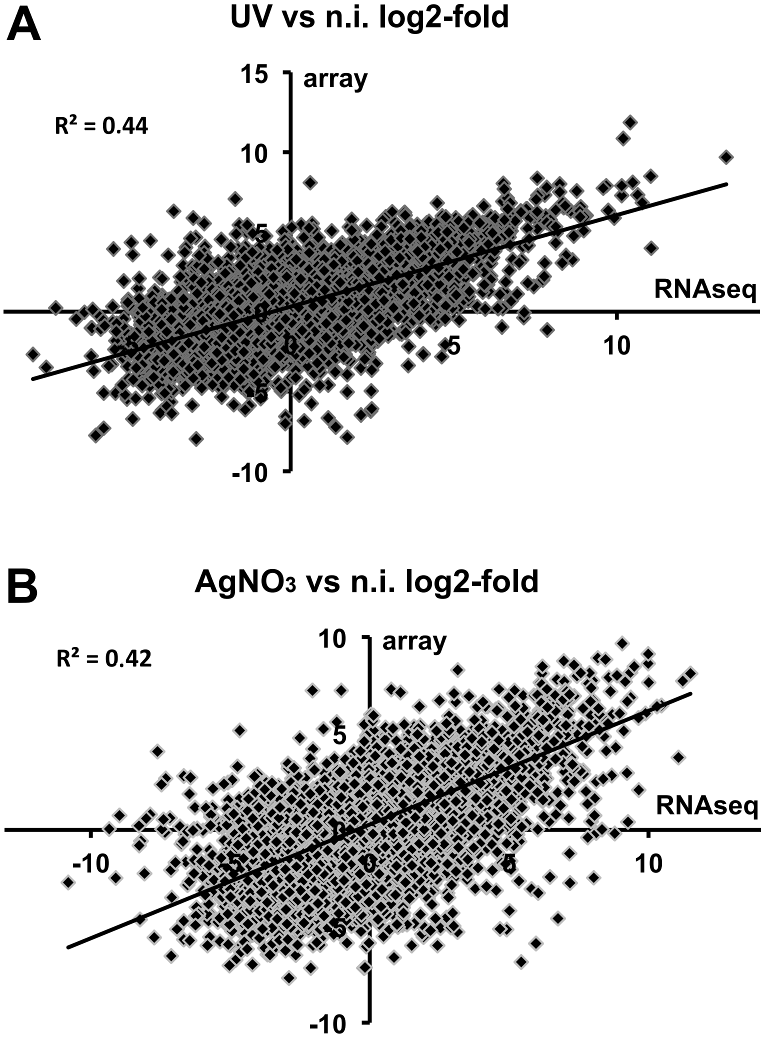

Supplemental Figure 3: Correlation of log-fold changes between RNAseq and array data after matching. A: UV versus not induced (n.i.). B: $AgNO_3$ versus n.i..
