# Supplementary material for: Substantial reprogramming of the Eutrema salsugineum (Thellungiella salsuginea) transcriptome in response to UV and silver nitrate challenge
Source: BMC Plant Biol. 2015 Jun 12;15:137. doi: 10.1186/s12870-015-0506-5 (PMC4464140; doi:10.1186/s12870-015-0506-5)
Supplement: Additional file 7: Figure S4. — UV- and AgNO3-responsiveness of genes differentially regulated in other studies. A: 81 drought and 59 cold regulated E. salsugineum genes (Wong et al. [32]) B: Identified putative orthologs of A. thaliana genes upregulated in response to both B. cinerea and oxidative stress (115 identified, 7 unchanged in response to UV or Ag+). [file 12870_2015_506_MOESM7_ESM.pdf]

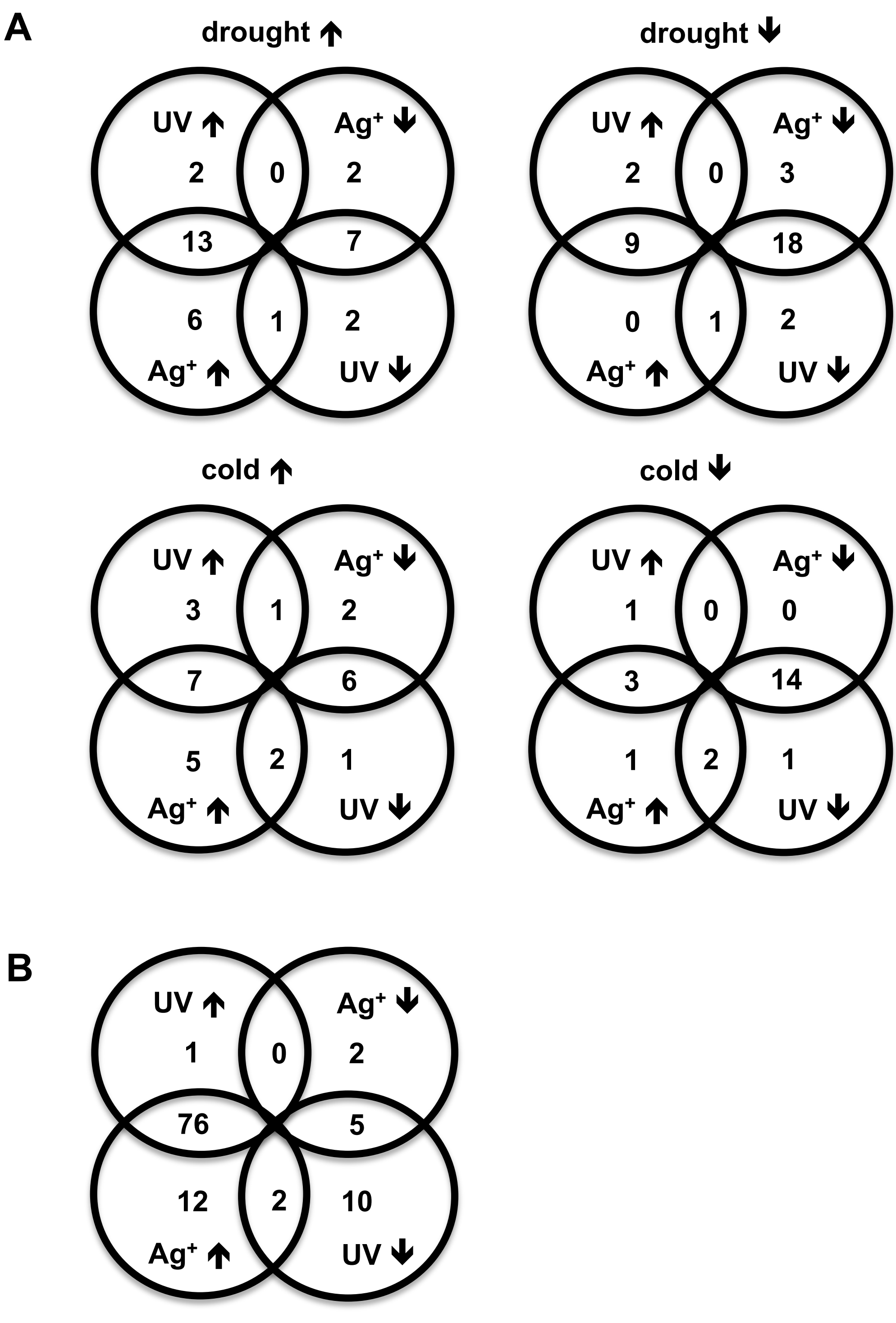

Supplemental Figure 4: UV- and $AgNO_3$-responsiveness of genes differentially regulated in other studies. **A:** 81 drought and 59 cold regulated *E. salsugineum* genes (Wong et al. Plant Physiol. 2006 140(4):1437-1450.) **B:** Identified putative orthologs of *A. thaliana* genes upregulated in response to both *B. cinerea* and oxidative stress (115 identified, 7 unchanged in response to UV or Ag^+)
